# Extracellular polymeric substances (EPS) producing and oil degrading bacteria isolated from the northern Gulf of Mexico

**DOI:** 10.1371/journal.pone.0208406

**Published:** 2018-12-06

**Authors:** Hernando P. Bacosa, Manoj Kamalanathan, Meng-Hsuen Chiu, Shih-Ming Tsai, Luni Sun, Jessica M. Labonté, Kathleen A. Schwehr, David Hala, Peter H. Santschi, Wei-Chun Chin, Antonietta Quigg

**Affiliations:** 1 Department of Marine Biology, Texas A&M University at Galveston, Galveston, Texas, United States of America; 2 Bioengineering Program, School of Engineering, University of California at Merced, Merced, California, United States of America; 3 Department of Marine Sciences, Texas A&M University at Galveston, Galveston, Texas, United States of America; 4 Department of Oceanography, Texas A&M University, College Station, Texas, United States of America; Universita degli Studi di Milano-Bicocca, ITALY

## Abstract

Sinking marine oil snow was found to be a major mechanism in the transport of spilled oil from the surface to the deep sea following the Deepwater Horizon (DwH) oil spill. Marine snow formation is primarily facilitated by extracellular polymeric substances (EPS), which are mainly composed of proteins and carbohydrates secreted by microorganisms. While numerous bacteria have been identified to degrade oil, there is a paucity of knowledge on bacteria that produce EPS in response to oil and Corexit exposure in the northern Gulf of Mexico (nGoM). In this study, we isolated bacteria from surface water of the nGoM that grow on oil or Corexit dispersant. Among the 100 strains isolated, nine were identified to produce remarkable amounts of EPS. 16S rRNA gene analysis revealed that six isolates (strains C1, C5, W10, W11, W14, W20) belong to the genus *Alteromonas*; the others were related to *Thalassospira* (C8), *Aestuariibacter* (C12), and *Escherichia* (W13a). The isolates preferably degraded alkanes (17–77%), over polycyclic aromatic hydrocarbons (0.90–23%). The EPS production was determined in the presence of a water accommodated fraction (WAF) of oil, a chemical enhanced WAF (CEWAF), Corexit, and control. The highest production of visible aggregates was found in Corexit followed by CEWAF, WAF, and control; indicating that Corexit generally enhanced EPS production. The addition of WAF and Corexit did not affect the carbohydrate content, but significantly increased the protein content of the EPS. On the average, WAF and CEWAF treatments had nine to ten times more proteins, and Corexit had five times higher than the control. Our results reveal that *Alteromonas* and *Thalassospira*, among the commonly reported bacteria following the DwH spill, produce protein rich EPS that could have crucial roles in oil degradation and marine snow formation. This study highlights the link between EPS production and bacterial oil-degrading capacity that should not be overlooked during spilled oil clearance.

## Introduction

The Deepwater Horizon (DwH) incident in 2010, the largest accidental oil spill in US history, released 4.9 million barrels of light Louisiana sweet crude oil into the Gulf of Mexico [1]. To mitigate the environmental impacts and enhance the biodegradation of oil, >2 million gallons of Corexit dispersant was applied to the surface and near the wellhead [2]. The spilled oil suffered different fates, in which massive amounts of oil were transported to the surface, reached the coastline and the marshlands, and deposited as marine oil snow (MOS) aggregates on the seafloor. Marine oil snow formation was one of the major processes that lead to the sedimentation of the oil to the seafloor [3,4,5].

Marine snow refers to ubiquitous particles in the ocean composed of organic and inorganic particles or aggregates (>0.5 mm), including minerals, detritus, bacteria, phytoplankton, zooplankton and feces [3,6,7]. Marine snow plays a crucial role in the transport of materials, such as oil and hydrocarbons, from the surface to the deep sea through gravitational settling. A large MOS formation event was observed in oil-contaminated waters of the northern Gulf of Mexico (nGoM) during the DwH spill [3]. The nGoM snow was found in difference sizes ranging up to several centimeters and appeared as compact, fluffy, or stringy mucus-like threads. Many of the floating snow materials were caught in the oil forming a web-like structure, and those that sunk were fluffy in appearance [3]. Marine snow appearing at the surface was formed mainly through the production of mucous webs by oil-degrading bacteria associated with the floating oil layers [3]. Marine snow is commonly formed by the coagulation and photo-aggregation [8] of this mucus with particles like cells, feces and minerals. However, bacteria alone can also form cm-sized and mucus-rich marine snow in the absence of particles [4]. When marine snow entraps oil droplets or oil components, it is referred to as marine oil snow (MOS). The transport of MOS to the deep sea through sediment and flocculent accumulation is referred to as marine oil snow sedimentation and flocculent accumulation or MOSSFA [5].

The mucus-like material acting as a precursor of aggregate formation, is mainly composed of extracellular polymeric substances (EPS) or simply exopolymers, which are high molecular weight exudates produced by bacteria and phytoplankton [7]. Chemically, EPS is composed largely of carbohydrates and proteins (75–90%), and in size continuum from dissolved to colloidal phases, including gels [7,9,10]. Higher protein to carbohydrate ratios are thought to exert control on EPS hydrophobicity, surface activity and therefore aggregate formation [11–14]. Marine bacteria produce EPS as a strategy for growth, adhering to solid surfaces, and to survive adverse conditions [15]. These adverse conditions include extremes of temperature, salinity, and nutrient availability, as well as petrochemically polluted areas. The released EPS can also influence the fate of oil and chemical dispersants in the ocean through emulsification, degradation, dispersion, aggregation and/or sedimentation [7]. When EPS or EPS-like materials bind with particles, marine snow can be determined as transparent exopolymer particles (TEP) by alcian blue staining [3,6]. The kinds of interactions of EPS or TEP depend on the type of bacteria, the kind of growth substrate, and the properties of the material produced.

Despite the growing evidence on the crucial role of EPS on the fate of spilled oil following the DwH, little has been known as to what specific bacterial taxa are responsible for the release of these exopolymers. The focus of previous studies was the succession of bacterial communities and bacterial genera associated with hydrocarbon degradation [16]. The aliphatic hydrocarbon degrading bacteria *Oceanospirillales* initially dominated the deep-sea plume, and succeeded by aromatic-degrading *Cycloclasticus*, *Pseudoalteromonas* and hydrocarbon-degrading generalists *Colwellia* [17–19]. High proportions of *Alteromonas*, *Marinobacter*, *Thalassospira*, *Bartonella*, *Rhodovulum*, and *Stappia* were found in oil mousses collected from the nGoM [20], but it is uncertain whether they degrade oil, produce EPS or both. Different bacterial genera dominated the oil-contaminated water depending on the degree of oil weathering, and other factors such as sunlight, temperature, nutrients, dispersant, and oil concentration [16,21,22]. As EPS-producing bacteria likely represent dominant taxa in an enriched community in the presence of oil, many of them could be effective oil degraders as well. For example, the composition of bacterial community in an oil-aggregate indicates a multifunctional assemblage of known oil degrading and potentially EPS producing members of *Gammaproteobacteria*, *Alphaproteobacteria*, *Bacteroidetes* and *Planktomycetes* [23]. Although community analysis gives a big picture of the bacterial composition and their potential, our understanding on the identity of key species involved in EPS production in oil polluted waters is limited. To date, only *Alteromonas* sp. strain TK-46(2) and three *Halomonas* have been reported in the nGOM that utilize oil and also produce EPS [24,25]. However, the composition of the EPS they produce in the presence of oil, particularly carbohydrates and proteins, and their abilities to degrade the alkanes and PAHs in oil have not been characterized. Moreover, it is still not clear how the presence of Corexit dispersant affects the abilities of these strains to produce EPS.

In this study, we report on the characterization of nine bacterial strains isolated from the nGoM that degrade oil and produce EPS. We found that the majority of isolates belong to the genus *Alteromonas*, and showed differential abilities to degrade oil and produce EPS both in the presence and absence of Corexit. The protein and carbohydrate content of the EPS they produced, hydrocarbon degradation abilities, and exoenzymes produced, were also characterized to develop a mechanistic understanding of the important interacting factors in marine snow production.

## Materials and methods

### Bacterial isolation

The bacteria were isolated from a mesocosm experiment conducted from May to June 2017 (16 days) to understand the long-term formation of marine oil snow. The glass mesocosm tanks (130-L) were filled with nGoM surface water collected offshore (Galveston, Texas) (29.2726°N, 94.8126°W). No specific permissions were required for sampling locations/activities as we were collecting water samples on public waters. There was no animal research or other activities requiring any kinds of permits.

Large volumes of a water accommodated fraction of oil (WAF) and a chemically enhanced WAF (CEWAF) containing both Macondo Surrogate oil and Corexit dispersant at a ratio of 1:20 (V/V), were produced in a baffled circulating tank system according to Wade et al. [26]. Briefly, water with oil was recirculated in the multi-chambered baffled circulating tank at 350 ml per min while water was being drawn from the bottom of the last chamber and pumped back to the surface of the first chamber. To allow adequate mixing, a magnetic stirrer was placed below the tanks that provide energy at 60 rpm. The stirrer was set to a speed such that there was only a shallow vortex when oil was added. Macondo surrogate oil obtained from the Marlin Platform Dorado (SO-20120211-MPDF-003), with similar properties to the DwH oil, was provided by BP. Six replicate mesocosm tanks were prepared for each treatment—Control, WAF, and diluted CEWAF (DCEWAF). Three tanks per treatment were sacrificed for sample collection after 4 d and the remaining tanks were used after 16 d of the experiment. The tanks were incubated at 19°C in a 12:12 light/dark cycle.

Bacterial isolation was only performed after 4 d of incubation by collecting 10 mL from each of three WAF and three DCEWAF tanks. Previous mesocosm experiments showed that oil degradation and EPS production occur within 4 d [7]. The samples from the same treatment were pooled together, thoroughly mixed, and serially diluted. An aliquot of each bacterial suspension (100 μl) was plated onto an agar plate (1.5%) made from sterile seawater with 0.817 g/L of Bushnell Haas medium (BHM) [27,28]. In order to isolate oil-degrading bacteria from WAF tanks and Corexit-degrading strains from DCEWAF tanks respectively, ~50 μl of WAF and DCEWAF was spread uniformly on plates with oil or Corexit as sole carbon sources, respectively. The plates were then incubated at 19°C in a 12:12 light/dark cycle for 7 d. Colonies were picked and transferred to test tubes containing 2 mL of nGOM seawater with BHM and 200 mg/L of oil or 10 mg/L Corexit [29]. When the growth was confirmed by microscopy after 4 d, the entire volume was transferred to 50 mL test tube containing 25 mL of the medium and corresponding oil and Corexit concentrations. About 100 isolates were screened at this stage. Those that produced mucus-like aggregates in 4 d were selected and sent out for DNA sequencing.

### DNA sequencing

To obtain adequate biomass, the isolates were grown in Marine Broth 2216 (Difco) overnight. Cells were harvested by centrifugation followed by extraction of genomic DNA using DNA Mini Prep GenCatch ^TM^ Blood &Tissue Genomic Mini-Prep Kit (Epoch Life Science, Inc). The nearly full-length 16S rRNA gene was amplified by PCR using primers 27F (AGAGTTTGATCCTGGCTCAG) and 1492R (GGTTACCTTGTTACGACTT). The PCR cycle parameters were 96°C for 5min, followed by 25 cycles of amplification (96°C for 30 s, 55°C for 30 s, 72°C for 30s) and a final extension at 72°C for 5 min. Sequencing was performed by Epoch Life Science, Inc (Missouri, TX) on Applied Biosciences 3730xl DNA Analyzer following established protocols.

### Phylogenetic analysis

PCR-amplified rRNA gene sequences were compared with previously deposited sequences using the RDP v10 Classifier (SSU rRNA) [30] and National Center for Biotechnology Information (NCBI) BLAST [31] nucleotide database (nt). Using SINA [32], the SSU rRNA gene sequences were aligned with sequences selected with the RDP Seqmatch (SSU rRNA gene sequences from isolates, ≥1200 bp of good quality) from the RDP pipeline [33]. A maximum likelihood tree of the SSU rRNA gene sequences (100 bootstrap replicates) was constructed using phyML v3.1 [34] with the best model [GTR model with a gamma distribution (+G), estimated rates of variation among sites and a proportion of invariable sites (+I)], as determined with jModelTest 2 [35].

### Incubation for EPS production

Isolates were pre-cultured in Marine Broth 2216 (Difco) overnight. The cells were then harvested by centrifugation and re-suspended in sterile seawater with 0.817 g/L BHM. An aliquot of the suspension (10 μL) was pipetted to the 96-well ELISA plates containing of seawater only, WAF, CEWAF, and Corexit. The WAF and CEWAF were prepared as described above but in small volumes (500 mL). The plates were incubated at 19°C for 4 days with a 12:12 light/dark cycle. After 4 d the cells were processed for protein, carbohydrates and DNA analysis. Each treatment was prepared in five replicate wells. All incubation experiments were performed once.

To assess the formation of visible aggregates, aliquots of the same suspension (500 μL) were inoculated in 20-mL sterile scintillation vials containing 10 mL of seawater, WAF, CEWAF and Corexit prepared as described above. The vials were incubated similarly to the ELISA plates. Mucus/aggregates were visible to the naked eye at ~1mm in size. Those formed were evaluated and characterized at the end of the 4-d experiment using the following grading system: 0-absent; 1-present; 2-more; 3- most.

### Analysis of EPS

#### Carbohydrates analysis—Enzyme Linked Lectin Assay (ELLA)

The supernatant containing secreted EPS was collected and briefly centrifuged at 1700xg (Megafuge 1.0R) to measure carbohydrates using the protocol adapted from Chen et al. [36] and Leriche et al. [37]. Briefly, the supernatant was incubated in a 96-well (Nunc MaxiSorp, VWR, CA, USA) plate overnight at 4°C. This was then washed with PBST (PBS + 0.05% Tween-20) and PBS and blocked with 1% BSA. The 96 well plate was washed again with PBST and PBS and incubated with lectin (Concanavalin A, ConA) (Sigma-Aldrich, MO, USA), conjugated to horseradish peroxidase (HRP; 5 mg/ml) (Sigma-Aldrich, MO, USA), at 37°C for 1 hr. The substrate, 3,3’,5,5’-Tetramethylbenzidine (TMB; Sigma-Aldrich, MO, USA), was added to each well at room temperature followed by H_2_SO_4_ (Sigma-Aldrich, MO, USA) in order to terminate the reaction. The optical density was measured at 450 nm by PerkinElmer VICTOR3 (MA, USA) [38].

#### Protein analysis

The protein in the EPS was analyzed using NanoOrange Protein Quantification Kit (ThermoFischer) following the manufacturer protocol. Briefly, 30 μL of the sample was diluted in 1X NanoOrange working solution followed by incubation at 95°C for 10 min. The plates were then allowed to cool at room temperature for 20 min. Fluorescence measurements were carried out on a spectrophotometer for 1 sec using excitation/emission wavelengths of 485/590 nm.

#### DNA analysis

The pelleted bacterial cells were analyzed using ZR-96 Quick-gDNA kit (ZYMO Research, CA, USA) following the manufacturer’s protocol. Briefly, 4× lysis buffer was used to break the cells, these were passed through a DNA binding column, and then elution buffer was used to collect the DNA whose concentration was measured by Nano Drop ND-1000 (Thermo, CA USA) [38]. The abundance of protein and carbohydrates in each well was normalized to respective DNA concentrations.

### Exoenzyme assays

Activities of five extracellular enzymes were measured on the bacterial isolates after the 4 d incubation. The procedures, described in Yamada and Suzumura [39], were followed for enzyme activity measurements. Briefly, the samples were incubated with the fluorogenic substrates at a final concentration of 0.2 mM followed by incubation at room temperature in the dark for 3 hours. 4-methylumbelliferyl-α-D-glucopyranoside and 4-methylumbelliferyl-β-D-glucopyranoside were used for α- and β- glucosidase respectively, while 4-methylumbelliferyl oleate was used for lipase amino-peptidase and 4-methylumbelliferyl phosphate was used for alkaline phosphatase, and leu-AMC-hydrochloride was used to assay leucine amino-peptidase. The addition of 0.4 M borate buffer solution adjusted at pH 8.0 for 7-amido-4-methylcoumarin (AMC)-tagged substrates and at pH 10.0 for 4-methylumbelliferyl (MUF)-tagged substrates stopped the reactions. Enzyme activity was then measured by fluorescence intensity at excitation/emission wavelengths (nm) of 380/440 (AMC) or 365/448 (MUF) using BioTek Cytation 5 imaging reader controlled by Gen5 (2.09) software. Heated samples were used as blank for these measurements.

### Oil degradation experiment

The biodegradation experiment was conducted using pre-combusted 120-mL amber bottles with MSO at the final concentration of 200 mg/L [21,22,29]. The isolates were pre-cultured overnight in seawater with 0.817 g/L of BHM. Bacterial cells were then harvested by centrifugation (3000xg for 15 min at 4°C), washed twice, and resuspended in the medium. The cells were then inoculated in the culture bottles to yield an initial density of 1 x 10^6^/mL. After adjusting the final volume to 50 mL, MSO was directly added to each bottle. To account for nonbiological losses, control bottles with no bacteria were similarly prepared. Triplicate bottles were prepared for each isolate. All bottles were incubated for 4 d in a 12:12 light/dark cycle with shaking (110 rpm). Bacterial cells were enumerated using a compound microscope. All treatments were prepared in triplicates.

### Hydrocarbon analysis

Hydrocarbon analysis was performed according to a previously established protocol [22,29,40]. Briefly, the samples were spiked with a mixture of deuterated standards (dodecane-d_26_, hexadecane-d_34_, naphthalene-d_8_, phenanthrene-d_10_ and pyrelene-d_12_) and extracted three times with 15 mL dichloromethane. The extracts were combined and passed through a chromatographic column with 20 g anhydrous sodium sulfate to remove excess water. Finally, the extracts were concentrated by rotary evaporator to 500 μl.

Alkanes, polycyclic aromatic hydrocarbons (PAH), and alkylated PAHs were analyzed using HP-6890 Series GC (Hewlett Packard) interfaced with an Agilent 5973 inert mass selective detector (MSD), and operated in a selective ion monitoring (SIM) mode. The hydrocarbons were resolved in the Agilent DB-5MS column (30-m long, 0.25-mm I.D., 0.25-μm thick). The operating conditions were as follows: 40°C for 1 min, ramped at 20°C/min to 180°C, ramped at 5°C/min to 300°C and held for 28 min. The detection limit of the instrument was 2.5 μg/L and the surrogate recoveries varied between 55–112%. The hydrocarbons were quantified using the deuterated standards. Reported concentrations of target analytes were recovery-corrected. The efficiency of biodegradation was computed relative to the residual concentration in the sterile control as mentioned elsewhere [41].

### Scanning electron microscopy

Samples for scanning electron microscopy (SEM) were first fixed using 4% paraformaldehyde, then washed with a phosphate-buffered saline (PBS) followed by deionized water rinse. Dehydration was completed by using 30, 50, 75, 95, and 100% methanol. A CO_2_ critical point dryer was used to remove any residual solvents. Finally, a thin layer of Au was deposited on these substrates. Images were then acquired using a FEI Quanta 200 ESEM system [42].

### Statistical analysis

The differences among the means of protein and carbohydrates ratios, enzyme activities, and hydrocarbon degradation, were analyzed by one-way analysis of variance (ANOVA) in PAST software package, V2.17 [43]. When a significant difference was obtained, the means were further tested by Tukey’s pairwise comparison.

### Nucleotide sequence accession numbers

The ca. 1400 bp sequences of the nine strains were deposited in the GenBank database with accession numbers MG214522 to MG214530.

## Results and discussion

### Bacterial identification and phylogeny

**Fig 1:**
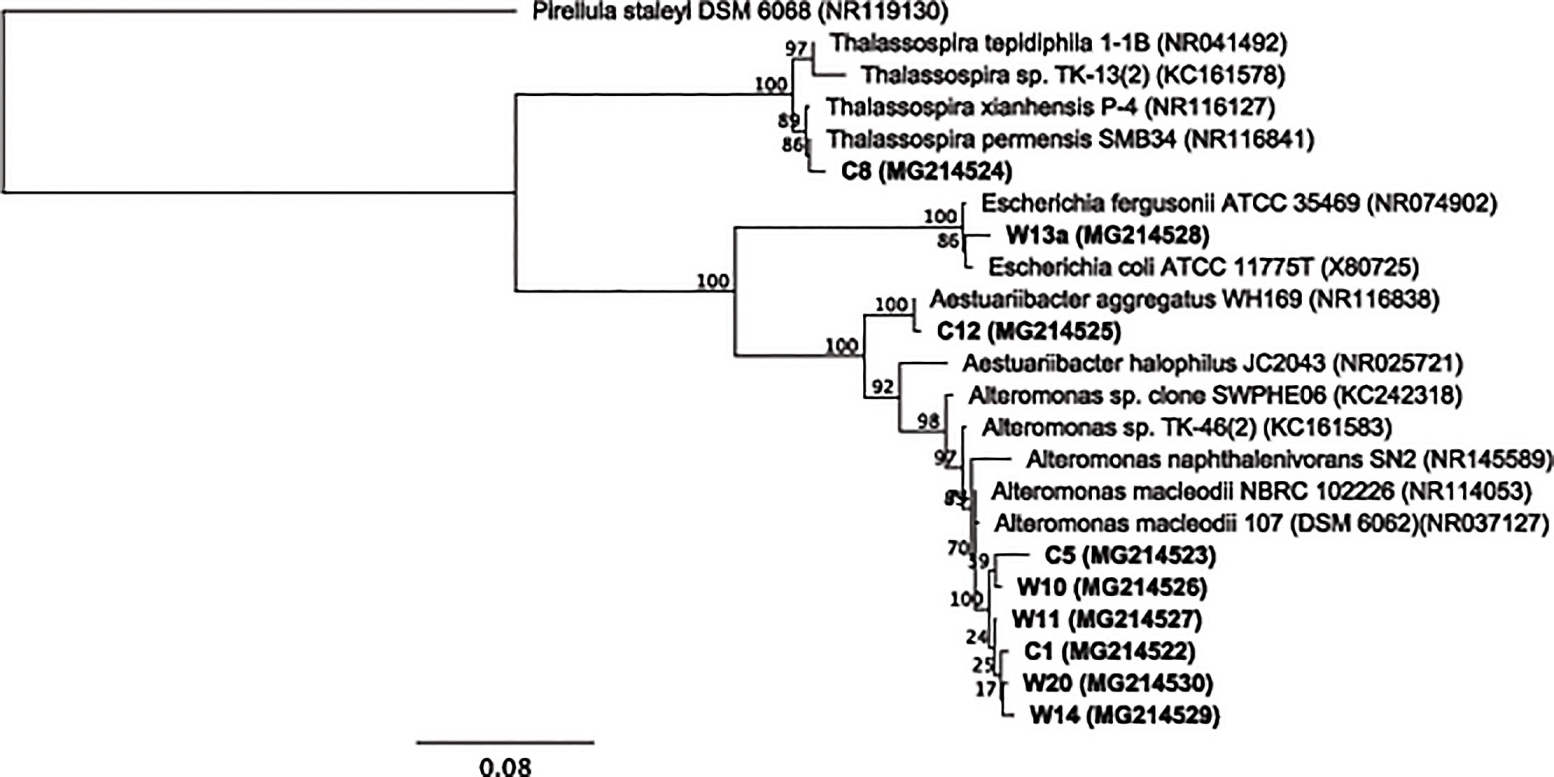
Phylogenetic tree (maximum likelihood: 100 bootstrap replicates) of the isolates, their closest relatives, and other strains isolated from the Gulf of Mexico.

Of the 100 strains isolated, nine were confirmed to have produced mucus-like aggregates in the presence of oil or Corexit as carbon sources. The analysis of 16S rRNA gene sequences revealed that these isolates all belong to phylum Proteobacteria and three classes (*Alteromonadales*, *Rhodospirillales*, and *Enterobacteriales*). The majority of the isolates were identified as members of the genus *Alteromonas* (strains C1, C5, W10, W11, W14, W20) (Table 1). The three other isolates were *Thalassospira* (strain C8), *Aestuariibacter* (strain C12), and *Escherichia* (strain W13a) All sequences shared a similarity of >97% to their closest relative in the GenBank and type strains (Fig 1).

**Table 1:** Genotypic characterization of the nine bacterial isolates.

Isolate	GenBank accession number	Sequence length (bp)	Source mesocosm	Carbon source	Closest relative in GenBank (accessed on Oct. 17, 2017)	Similarity
C1	MG214522	1442	DCEWAF	Corexit	*Alteromonas macleodii* NBRC 102226 (NR114053)	97.6%
C5	MG214523	1445	DCEWAF	Corexit	*Alteromonas macleodii* NBRC 102226 (NR114053)	98.7%
C8	MG214524	1407	DCEWAF	Corexit	*Thalassospira xianhensis* P-4 (NR116127)	97.6%
C12	MG214525	1440	DCEWAF	Corexit	*Aestuariibacter aggregatus* WH169 (NR116838)	99.4%
W10	MG214526	1440	WAF	Oil	*Alteromonas macleodii* 107 (NR037127)	98.6%
W11	MG214527	1438	WAF	Oil	*Alteromonas macleodii* NBRC 102226 (NR114053)	98.7%
W14	MG214529	1437	WAF	Oil	*Alteromonas macleodii* 107 (NR037127)	98.9%
W20	MG214530	1435	WAF	Oil	*Alteromonas macleodii* 107 (NR037127)	98.7%
W13a	MG214528	1443	WAF	Oil	*Escherichia fergusonii* ATCC 35469 (NR074902)	99.5%

The closest relative of the *Alteromonas* isolates is *Alteromonas macleodii*. These isolates have less than 98% sequence similarity to the known PAHs-degrading *Alteromonas naphthalenivorans* SN2 [44]. *Alteromonas* dominated in the subsurface plume of the nGoM after the well was shut in [19], and was abundant in the surface oil mousses [20]. *Alteromonas* dominated in nGoM surface water with oil and both oil and dispersant when incubated under natural sunlight [21]. An oil-degrading and EPS-producing *Alteromonas* sp. strain TK-46(2) was isolated from surface oil slicks in the nGoM during the DWH spill [24,45]. This strain was isolated using pure hydrocarbon compounds that include hexadecane, naphthalene, and phenanthrene [45]. However, the *Alteromonas* sequences in this study have a similarity range of 97.0–98.9% to strain TK-46(2), and 96.5–97.8% similar to *Alteromonas* SIP clone SWNAP06 [45]. Clone SWNAP06 was the representative clone of the *Alteromonas* that assimilated naphthalene in DNA-stable isotope probing experiment using surface water less than one kilometer away from the DWH site. This suggests that the oil-degrading and/or EPS-producing *Alteromonas* in the nGoM could be more diverse that previously thought. Moreover, unlike single hydrocarbon compounds used by Gutierrez et al. [45], we used oil in isolating these bacteria that is more representative of the actual pollutant.

*Thalassospira* was also abundant in oil mousses collected after the spill [20] and comprised up to 30% of total community in incubations containing both oil and Corexit [21]. At the 16S rRNA gene level, the *Thalassopira* (C8) in this study is distantly related (95.5%) to *Thalassospira* sp. TK-13(2) previously isolated from the nGoM along with *Alteromonas* TK-46(2) [45]. Both *Alteromonas* and *Thalassospira* in the nGoM have been reported to have genomic potentials for PAH degradation [46]. *Escherichia* and *Aestuariibacter* were not previously reported to be associated with oiled samples from the nGoM following the DWH spill. It could be attributed to the sampling location as water samples in this study was obtained near the coast (8 km). Alkane and PAHs-degrading *Escherichia* was isolated from the coastal area in India [47]. *Aestuariibacter* OTU3 represented 30–34% of the total community in CEWAF treatments in mesocosm studies using coastal surface water of the nGoM amended with oil [48].

### EPS production

Visible aggregates differed–in both quantity and quality—across treatments and isolates. In general, controls had the least visible aggregates (score of 0.94), while Corexit treatments produced the most aggregates (score of 2.39) (Table 2). On average, WAF had more aggregates than the Control, and CEWAF had more than the WAF. Moreover, all “C” strains, which were isolated using Corexit, produced aggregates in Corexit treatment. Similarly all “W” isolate yield visible aggregates in the presence of WAF. The EPS also produce a matrix that appeared to glue many bacterial cells together (S1 Fig).

**Table 2:** Visible aggregates (mucus) observed after 4 d of incubation in control, WAF, CEWAF, and Corexit. The values represent the average of duplicate samples. (0-none; 1-present; 2-more; 3- most).

Isolate	Control	WAF	CEWAF	Corexit
C1	1	1	2	2
C5	0	1	0	2
C8	1.5	0	2	1.5
C12	1	1	0	2
W10	0	1.5	2	3
W11	2	3	3	3
W14	0	1	1	3
W20	2	1	0	3
W13a	1	2	2	2
Mean	0.94^b^	1.28^b^	1.33^b^	2.39^a^

**Fig 2:**
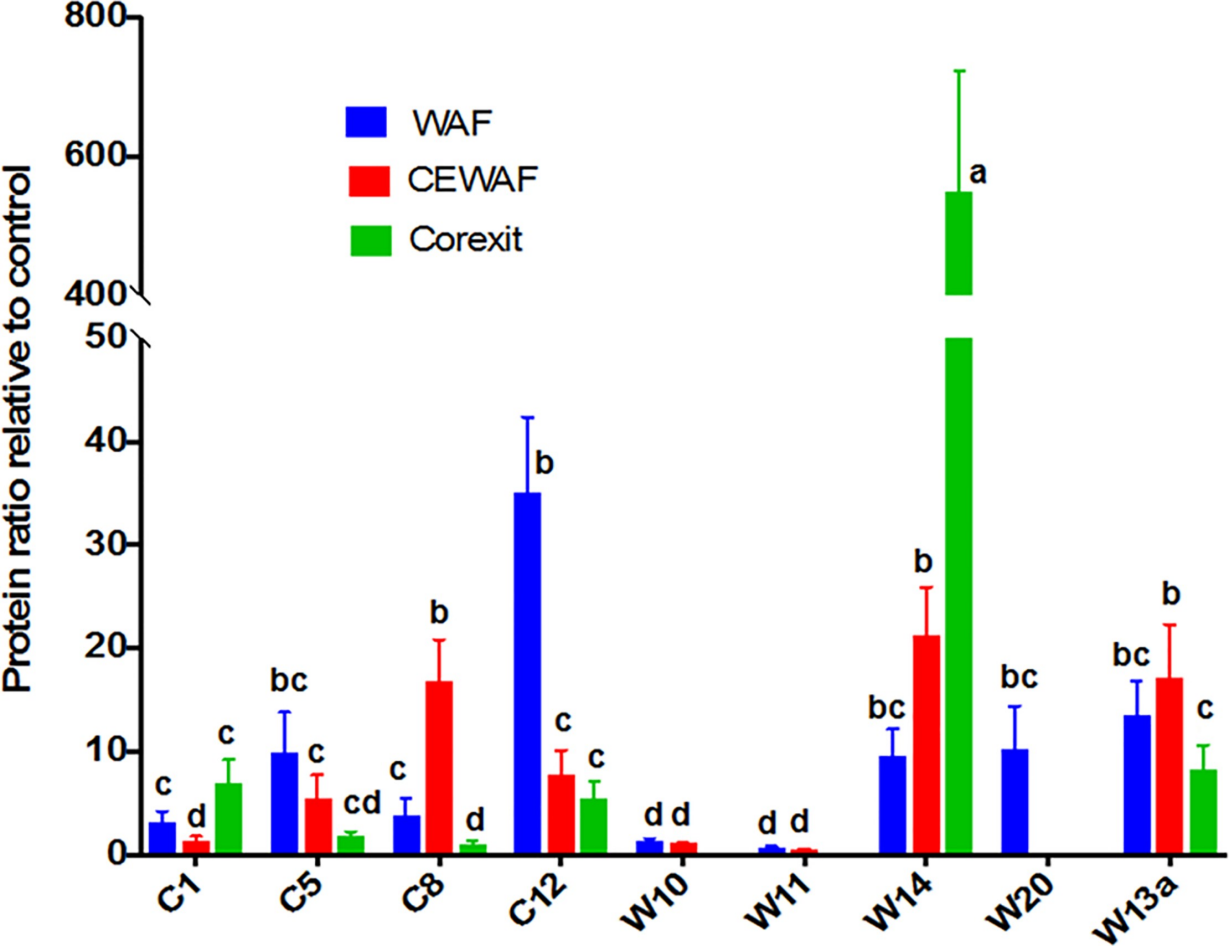
Production of protein by the isolates in WAF, CEWAF and Corexit after 4 d of incubation. All values are normalized to Control. Error bars represent the standard deviation of the replicates. Different letters above bars indicate significant differences among treatments (P <0.05).

**Fig 3:**
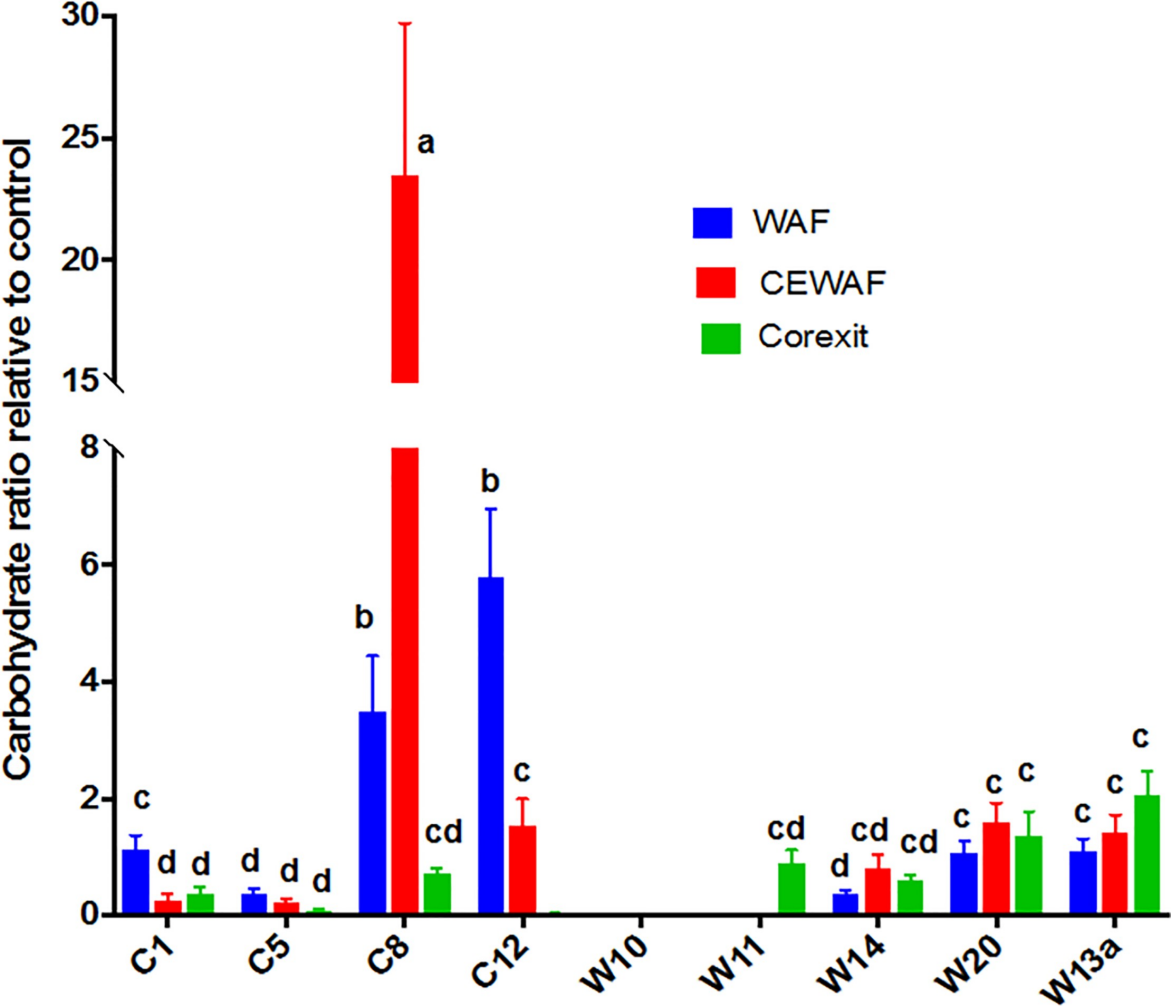
Production of carbohydrates by the isolates in WAF, CEWAF and Corexit after 4 d of incubation. All values are normalized to Control. Error bars represent the standard deviation of the replicates. Different letters above bars indicate significant differences among treatments (P <0.05).

EPS are primarily composed of proteins and carbohydrates, and to a lesser extent nucleic acids and other cellular products [7]. The protein and carbohydrate content of the EPS varied with treatment and strain (Figs 2 and 3, respectively). The addition of oil and Corexit remarkably increased the protein content of EPS. WAF and CEWAF exposed bacteria produced 10 and 9 times more protein than the Control, respectively (Fig 2). Corexit treatments elicited a 5-fold increase in protein, except for W14, which had 550x greater protein than the corresponding control. W14 also manifested a high increase of protein in WAF (10x) and CEWAF (21x). C12 yielded the highest protein increase in WAF treatments (35x). While protein content in the extracellular matrix was higher than the control, this was not the case for carbohydrates (Fig 3). With the exception of isolates C8 and C12, the carbohydrate content of EPS produced by bacteria in WAF treatments was only 0.50x of the control, while that of CEWAF and Corexit was on the average 0.63x and 0.77x than that of the control, respectively (Fig 3). Only C8 (3x in WAF and 23x in CEWAF) and C12 (6x in WAF) showed a remarkable increase in carbohydrate production relative to the controls. Microbial EPS composition is known to be species specific as well as treatment dependent [14,49,50].

The type of growth substrate is a major factor that influences EPS production [51,52]. In the presence of toxic substances including heavy metals and organic compounds, bacterial cells produce more EPS to protect themselves from such a harsh environment [51,53]. Under these toxic conditions, bacteria in the mixed-culture systems such as biofilms, activated sludge and anaerobic granular sludge commonly produce EPS that are primarily composed of proteins [54,55]. This is in contrast to most EPS produced in pure culture studies that were mainly polysaccharides [52,56]. Here, we showed for the first time that the isolates incubated with oil and/or Corexit produced EPS with higher protein content. The role of high protein in more adverse condition is not yet clear, but likely associated to increased aggregation to protect the cells.

In our previous mesocosm experiment using the natural microbial communities (phytoplankton and bacteria) in the Gulf of Mexico, the presence of Corexit appears to be a key driver in the increase of protein in EPS (CEWAF>DCEWAF>WAF>Control) [57]. Although bacteria naturally produce EPS enriched in protein [58], here we showed that the presence of oil and Corexit generally increase the protein content EPS produced by bacteria. However, as to whether Corexit induces higher protein production than oil depends on the kind of bacteria. Only *Alteromonas* W14 showed an increasing protein content with increasing Corexit concentration, whereas *Aestuariibacter* C12 showed the opposite pattern.

### Extracellular enzyme activity

**Fig 4:**
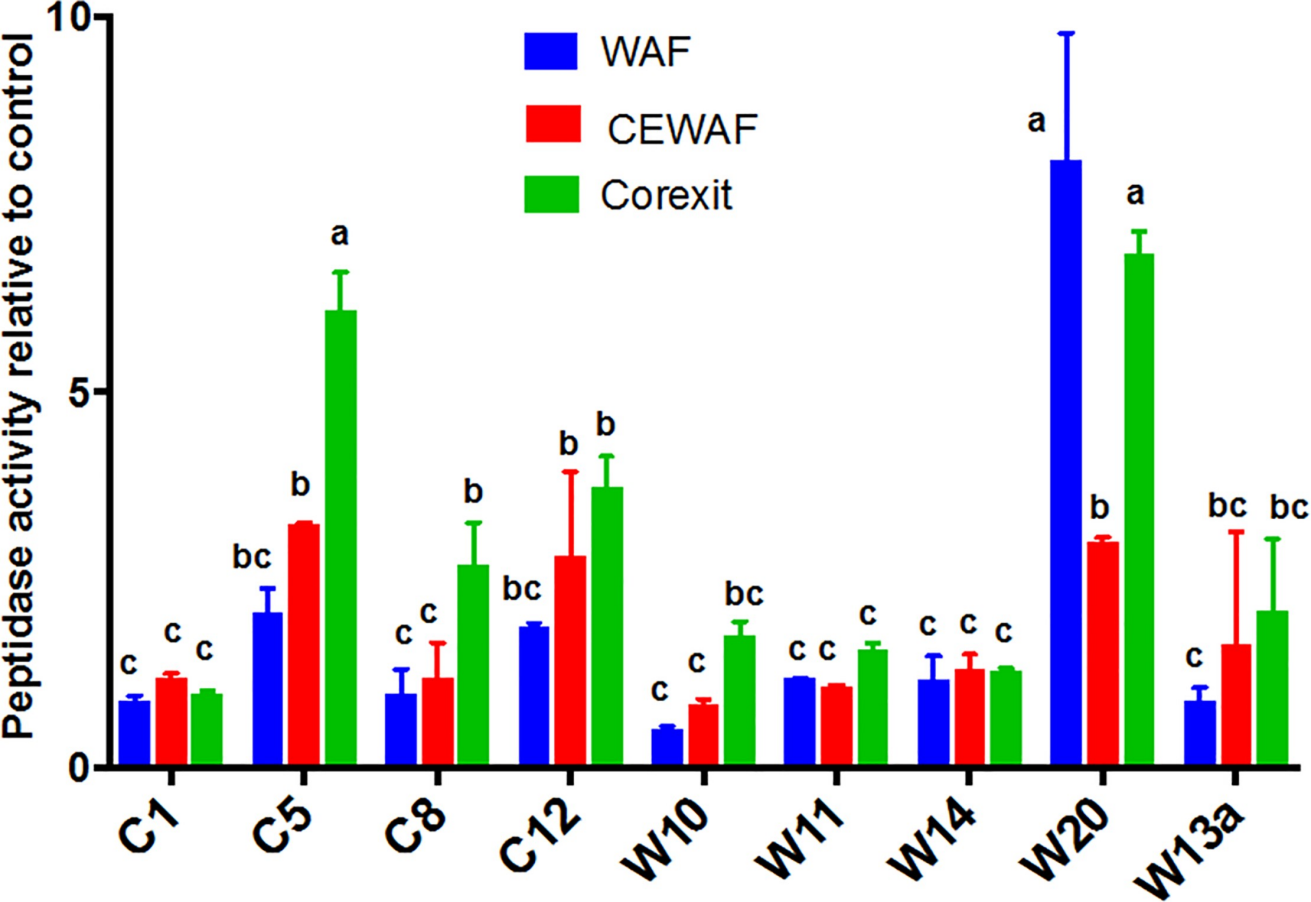
Leucine amino-peptidase activity in WAF, CEWAF and Corexit after 4 d of incubation with the bacterial isolates. The values are ratios relative to Control. Error bars represent the standard deviation of the replicates. Different letters above bars indicate significant differences among treatments (P <0.05).

**Fig 5:**
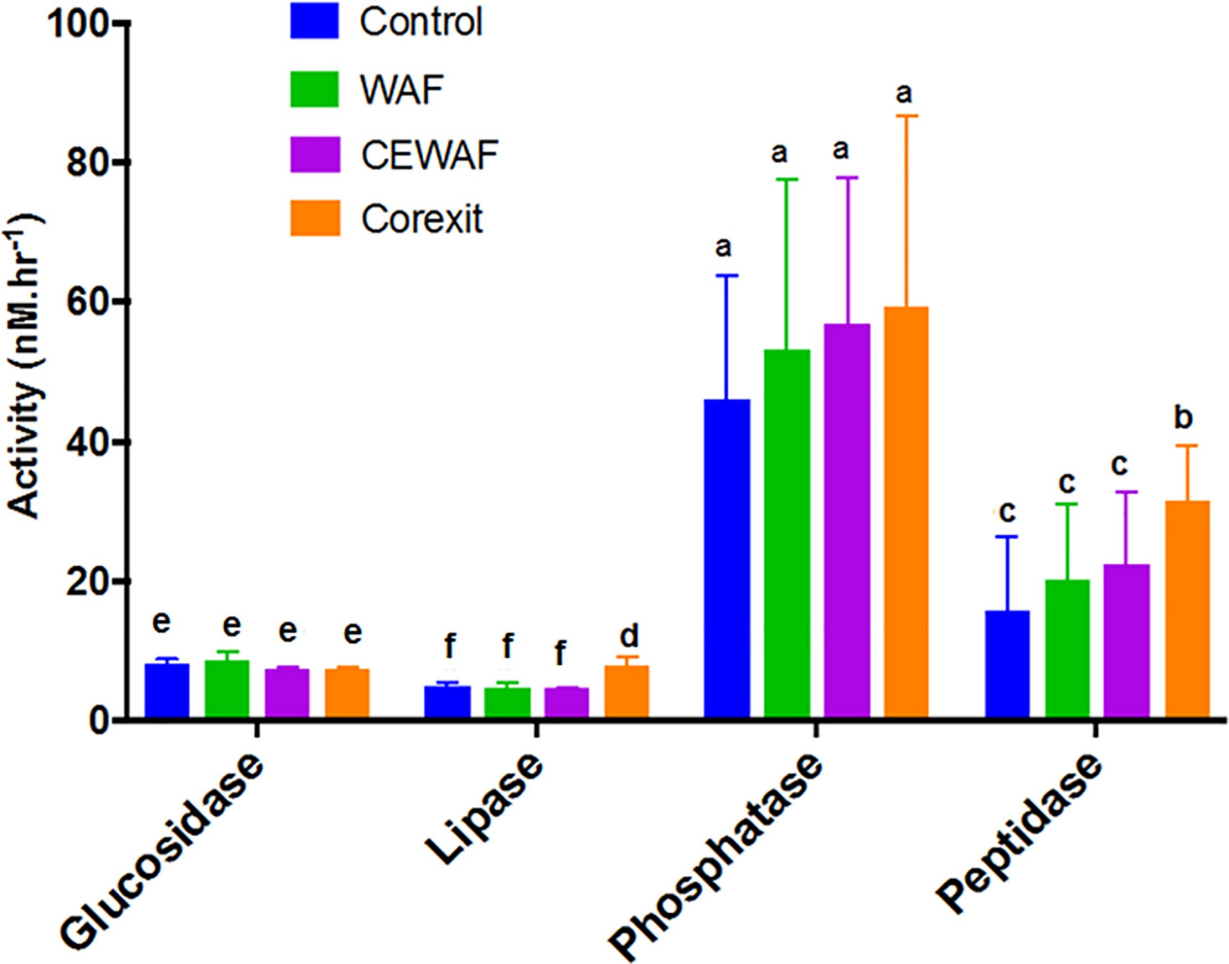
Average enzyme activities (± SD) of glucosidase, lipase, alkaline phosphatase, and leucine amino-peptidase in Control, WAF, CEWAF and Corexit incubated with the nine isolates. Error bars represent the standard deviation of the replicates. Different letters above bars indicate significant differences among treatments (P <0.05).

Many isolates showed an increasing trend in leucine amino-peptidase activity from Control to Corexit (Control<WAF<CEWAF<Corexit), with Corexit having the highest activity (Fig 4). C5, C12, W20 and W13a showed enhanced activity in the presence of oil and Corexit. In C5 and C12, peptidase activity was 2x and 3x higher in WAF and CEWAF compared to controls, whereas in the presence of Corexit the activity was 4–6 times higher. In W13a, leucine amino-peptidase was twice higher in CEWAF and Corexit. However, W20 showed the highest in WAF (8x) and Corexit (7x), while that of CEWAF was only 3x higher. Taken together, leucine amino-peptidase in Corexit was significantly higher than the rest of the treatments, similar to lipase (Fig 5; S2 Fig). When natural bacterial communities from the deep-sea were amended with oil and/or Corexit, the leucine amino-peptidase activity was also highest in Corexit only, followed by CEWAF, WAF and Control [59], overlapping with the dominance of *Colwellia* and *Marinobacter*. Here, we showed that this pattern is also exhibited by *Aestuariibacter* and some *Alteromonas* in surface water of the nGoM.

The increase in leucine amino-peptidase activity also corresponded to an increase in alkaline phosphatase activity (S3 Fig), consistent with our previous findings [60]. Most of these isolates also showed an increase in the protein content of EPS by up to 5 and 20 folds (Fig 2) suggesting that the increased peptidase activity is related to the increase in the protein content of the EPS of most bacteria. However, this is not the case for other bacteria. For example, there is no increase in peptidase in W14 even though there was 10 to 500 fold increase in protein EPS.

**Fig 6:**
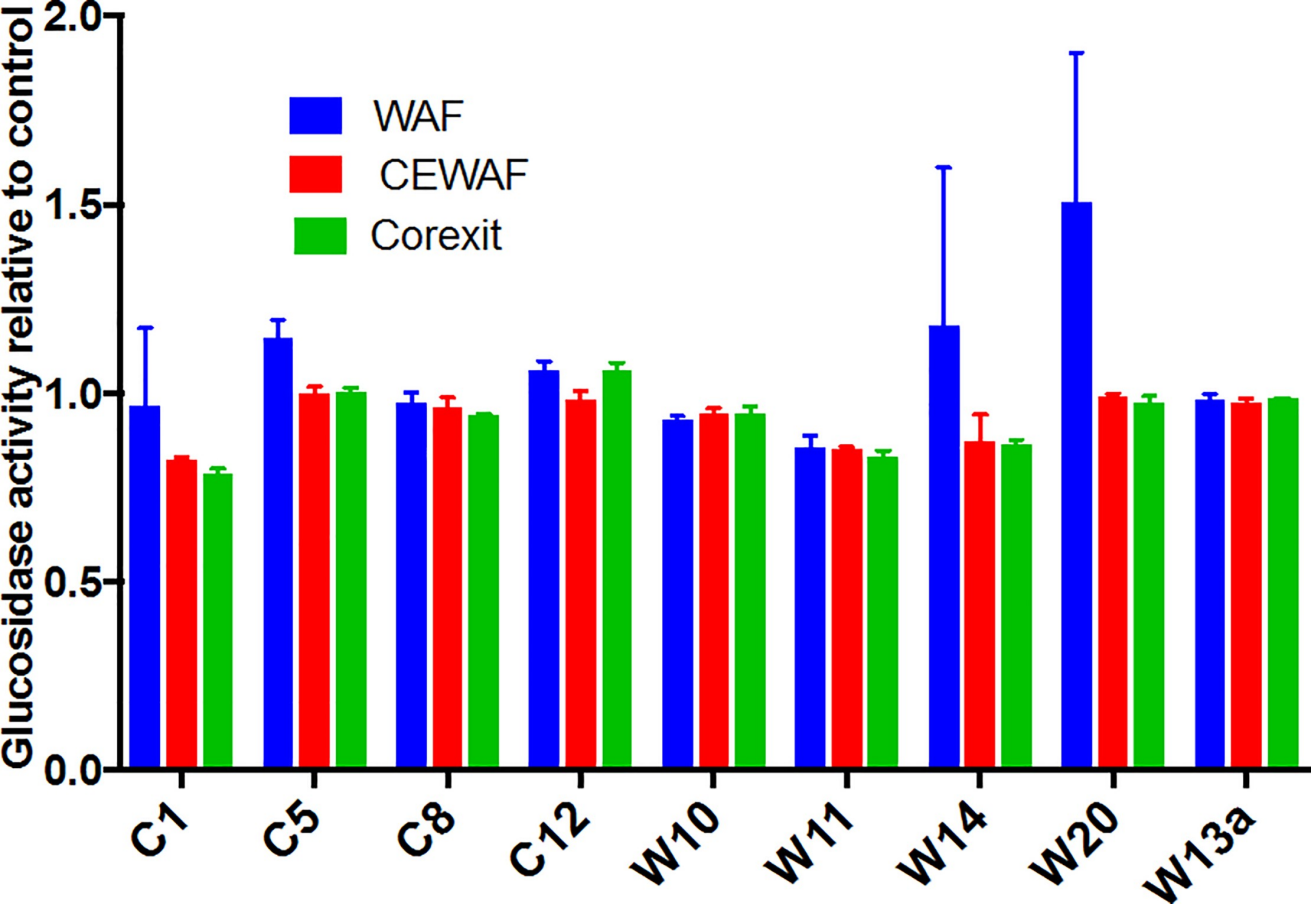
Glucosidase activity in WAF, CEWAF and Corexit after 4 d of incubation with the nine bacterial isolates. The values are ratios relative to Control. Error bars represent the standard deviation of the replicates. There was no significant difference among the treatments (P>0.05).

Glucosidases (shown here as sum of α and β glucosidase) are group of enzymes that bacteria produce to cleave at α and β linkages of carbohydrates [61]. There was no appreciable increase in glucosidase activity with the addition of oil and Corexit suggesting that bacteria were not utilizing the carbohydrates components of the EPS (Fig 5; Fig 6). Unlike protein, there was no remarkable increase in the carbohydrates content of EPS in the presence of oil and Corexit. It is possible that a significant increase in glucosidase activity can be achieved only with a higher increase in the carbohydrates content of the EPS such as when phytoplankton is present [62,63].

### Oil degradation

**Fig 7:**
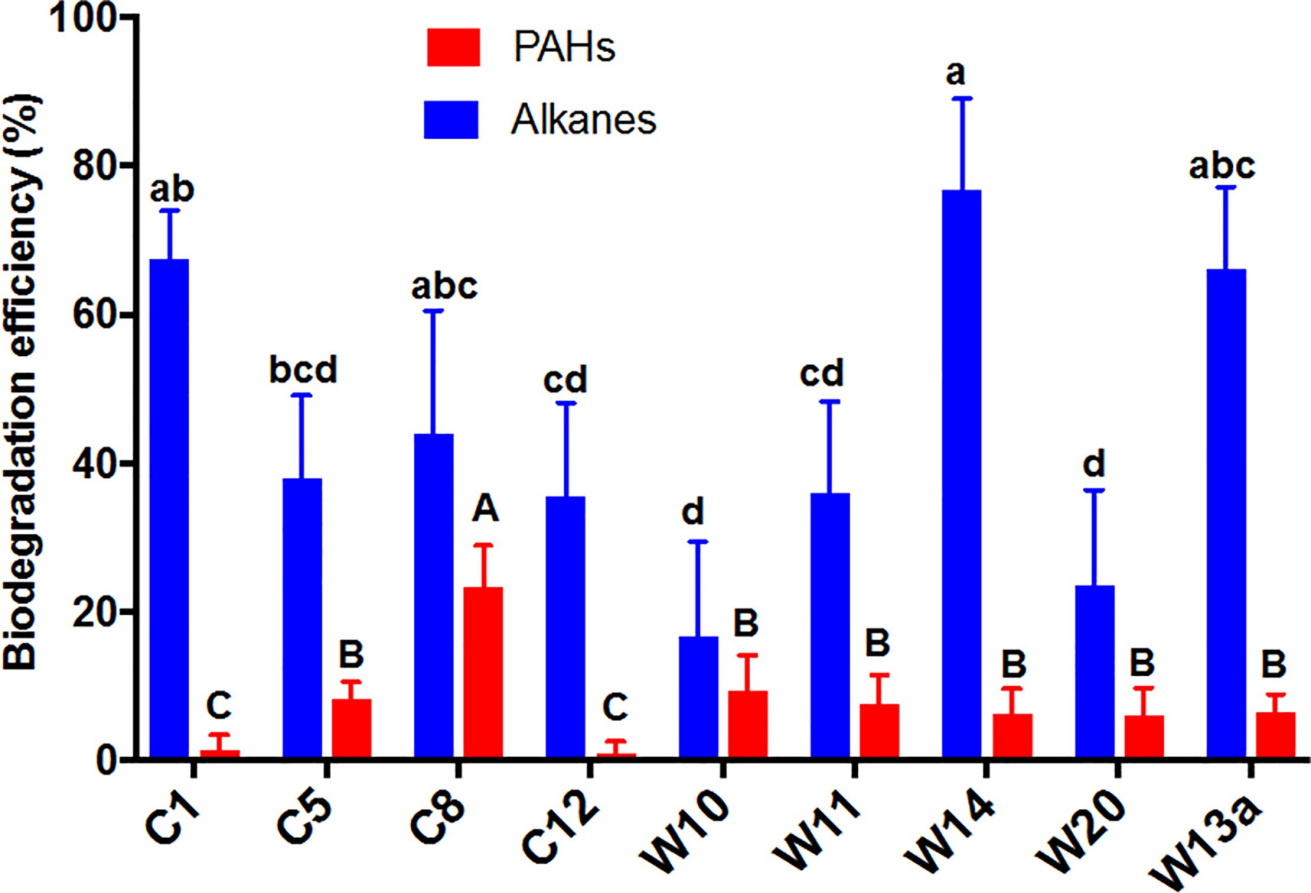
Degradation of *n*-alkanes and PAHs in oil by the bacterial isolates after 4 d of incubation. Error bars represent the standard deviation of three replicates. Biodegradation efficiency is percent degraded relative to abiotic control. Error bars represent the standard deviation of the replicates. Different letters above bars indicate significant differences among treatments (P <0.05).

The isolates degraded the oil at different rates during the four day incubation period (S4 Fig and S5 Fig). Alkanes were preferably degraded (16.8–76.9%) over the more toxic and recalcitrant PAHs (0.90–23.3%) (Fig 7), a typical biodegradation pattern [27,28,41,64,65]. W14 was the most effective degrader (76.9%, P<0.01) followed by W13a and C1 degrading more than 65% of alkanes. While W10 and W20 degraded only about 20% of alkanes, the remaining isolates degraded 35–45% of total alkanes. Interestingly, the six *Alteromonas* isolates have different abilities to utilize alkanes. Only C8 showed a significicantly degradation of PAHs (23.3%, P<0.01), while C1 and C12 hardly degraded these compounds (<2%). Most of the isolates were able to oxidize 6–9% of total PAHs. Although C1, C5, C8, and C12 were initially isolated and cultured using Corexit, these strains also use oil as carbon source.

Even though *Thalassospira* had been identified to be associated with microbial communities in oil-contaminated waters of the Gulf of Mexico [20,21,46,66,67] it is still uncertain what hydrocarbons does it degrade. For the first time we showed here a direct evidence of its abilities to degrade alkanes and PAHs (2 and 3 rings). Our findings further revealed that *Thalassopira* is an effective degrader of both alkanes and PAHs, contrary to previous claims of its greater role in the degradation of PAHs only [46,68]. *Alteromonas* has been reported as an important bacteria class that played an important role in the fate of oil [19,20,69]. Here we showed that *Alteromonas* in the GoM are effective in degrading the alkanes in oil but not PAHs. Our recent work also revealed that *Alteromonas* from the nGoM was abundant within first 5-d of incubation when the light hydrocarbons in crude oil were rapidly degraded [29]. We further revealed that all of these *Alteromonas* strains from the Gulf utilized mainly the naphthalenes in oil but not the 3-4-ring PAHs, an evidence of their dominance within the earlier stage of our previous incubation experiment [29]. This suggests that the abundance of *Altermonas* in oil-contaminated surface waters of the nGoM could be stimulated by the presence of fresher not by degraded oil.

## Conclusions

We successfully isolated nine bacterial strains from the surface waters of the nGoM that all produce EPS and degrade hydrocarbons. Most of the isolates belong to the genera *Alteromonas*, as well as *Thalassospira*, *Aestuariibacter*, and *Escherichia*. These bacteria produce protein rich EPS when exposed to oil and/or Corexit dispersant. Moreover, peptidase activity was enhanced with the exposure of these bacteria to oil and Corexit. The presence of Corexit appears to further enhance the production of EPS by bacteria. Our results support the previous findings [48] that the bloom of *Alteromonas* in mesocosm tanks could have resulted in a remarkable production of EPS. Moreover, the predominance of *Alteromonas* in this study and environmental samples and its ability to produce EPS and degrade hydrocarbons provides clues about their critical roles in marine snow formation during the DWH spill. Overall, we showed that hydrocarbon-degrading bacteria can also be effective producers of EPS that play critical roles in the emulsification and dispersion of oil droplets, and aggregation and sedimentation of oil with other particles to the deep sea.

## Supporting information

S1 Fig Image of isolate W14 under the scanning electron microscope (SEM) with its EPS produced by exposure to WAF. (PDF)

S2 Fig Lipase activity in WAF, CEWAF and Corexit after 4 d of incubation with the bacterial isolates. The values are ratios relative to Controls.
(PDF)

S3 Fig Alkaline phosphatase activity in WAF, CEWAF and Corexit after 4 d of incubation with the bacterial isolates. The values are ratios relative to Controls.
(PDF)

S4 Fig Concentration of *n*-alkanes in control and bottles inoculated with bacterial isolates after 4 d of incubation. Error bars represent the standard deviation of three replicates.
(PDF)

S5 Fig Concentration of PAHs in control and bottles inoculated with bacterial isolates after 4 d of incubation. Error bars represent the standard deviation of three replicates.
(PDF)
