# Supplementary figures and images for: Extracellular polymeric substances (EPS) producing and oil degrading bacteria isolated from the northern Gulf of Mexico
Source: PLoS One. 2018 Dec 6;13(12):e0208406. doi: 10.1371/journal.pone.0208406 (PMC6283562; doi:10.1371/journal.pone.0208406)

**S1 Fig.**

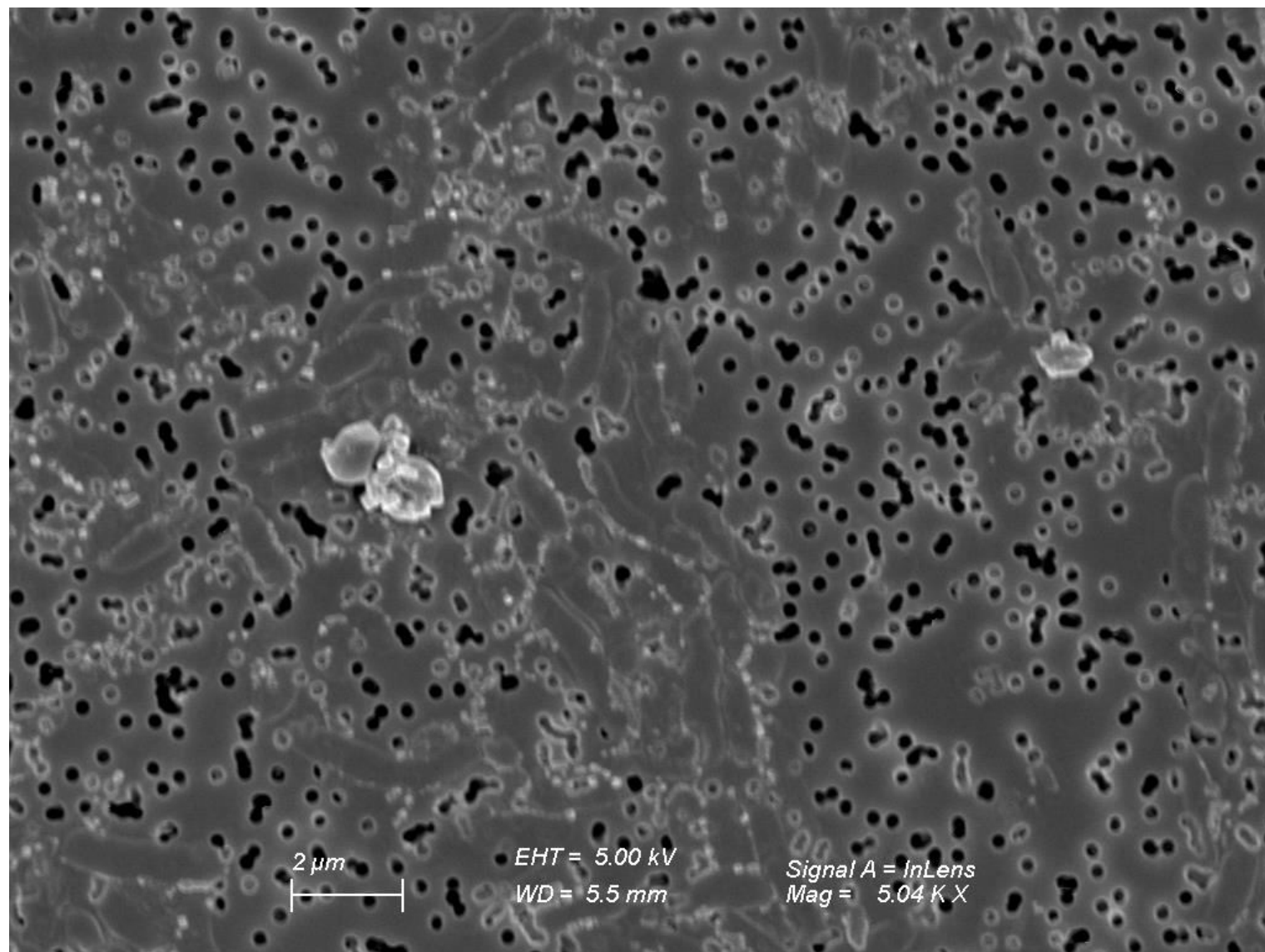

Supplement: S1 Fig — (PDF) [file pone.0208406.s001.pdf]

**S2 Fig.**

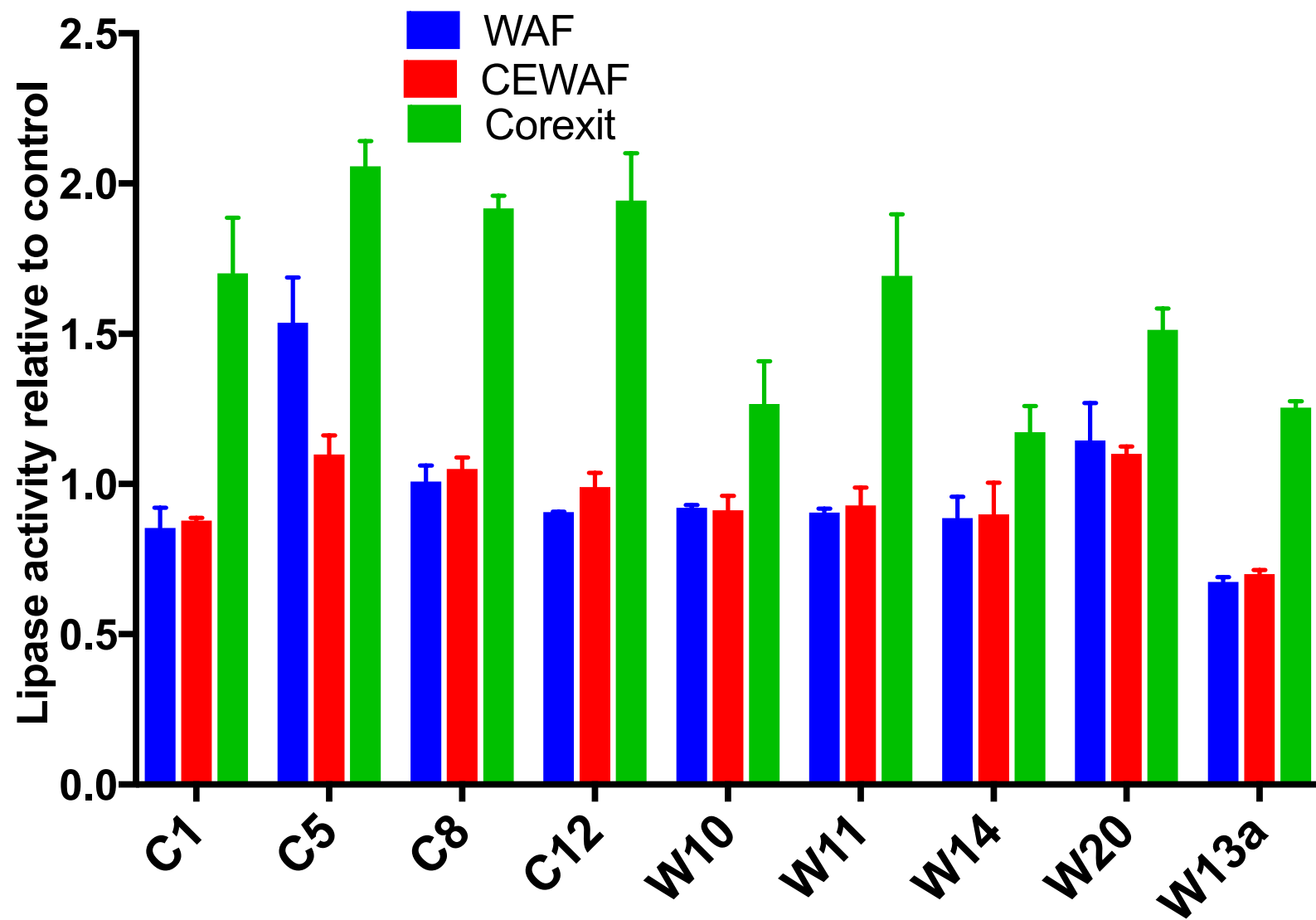

Supplement: S2 Fig — The values are ratios relative to Controls. (PDF) [file pone.0208406.s002.pdf]

S3 Fig.

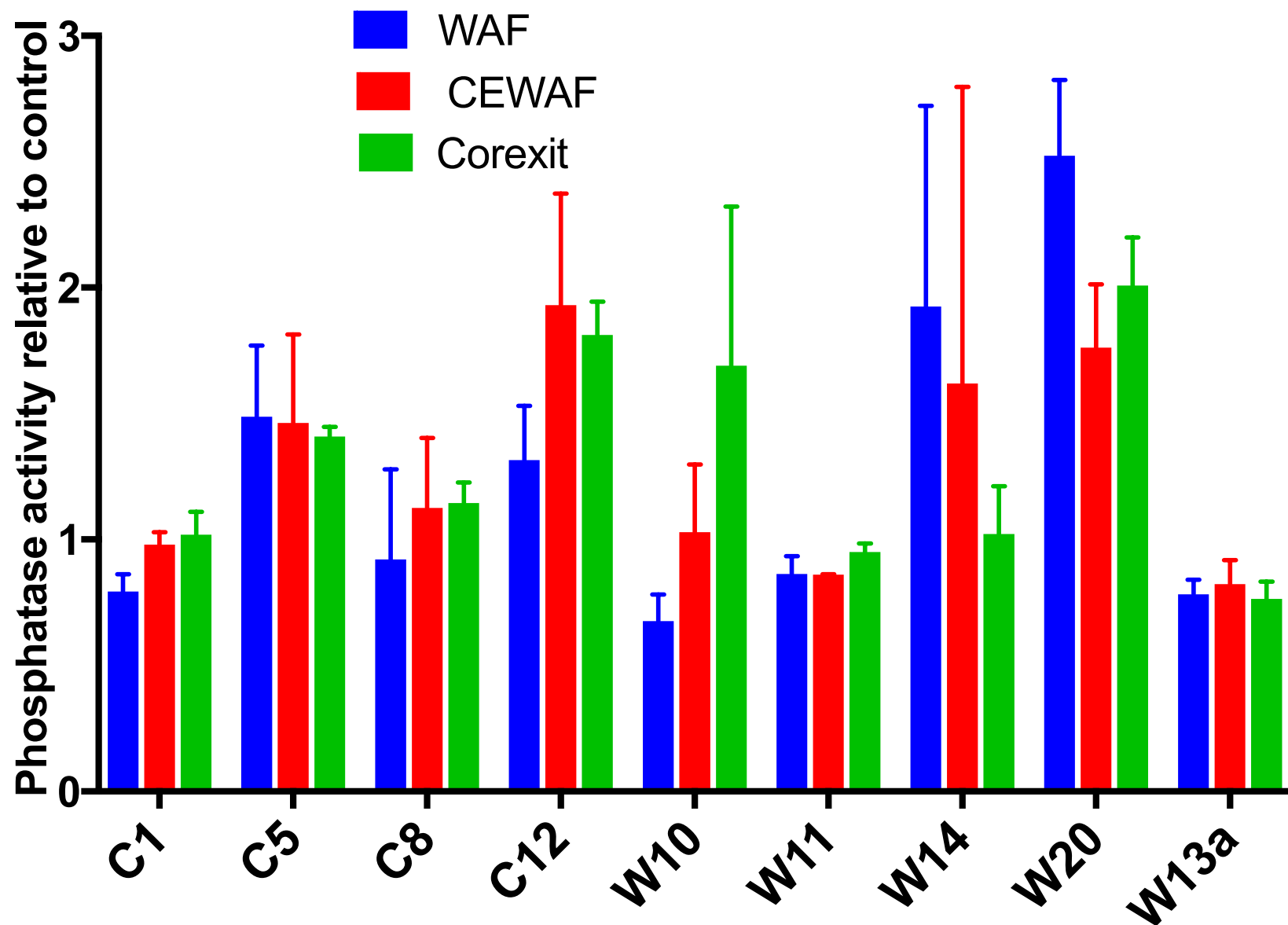

Supplement: S3 Fig — The values are ratios relative to Controls. (PDF) [file pone.0208406.s003.pdf]

**S4 Fig.**

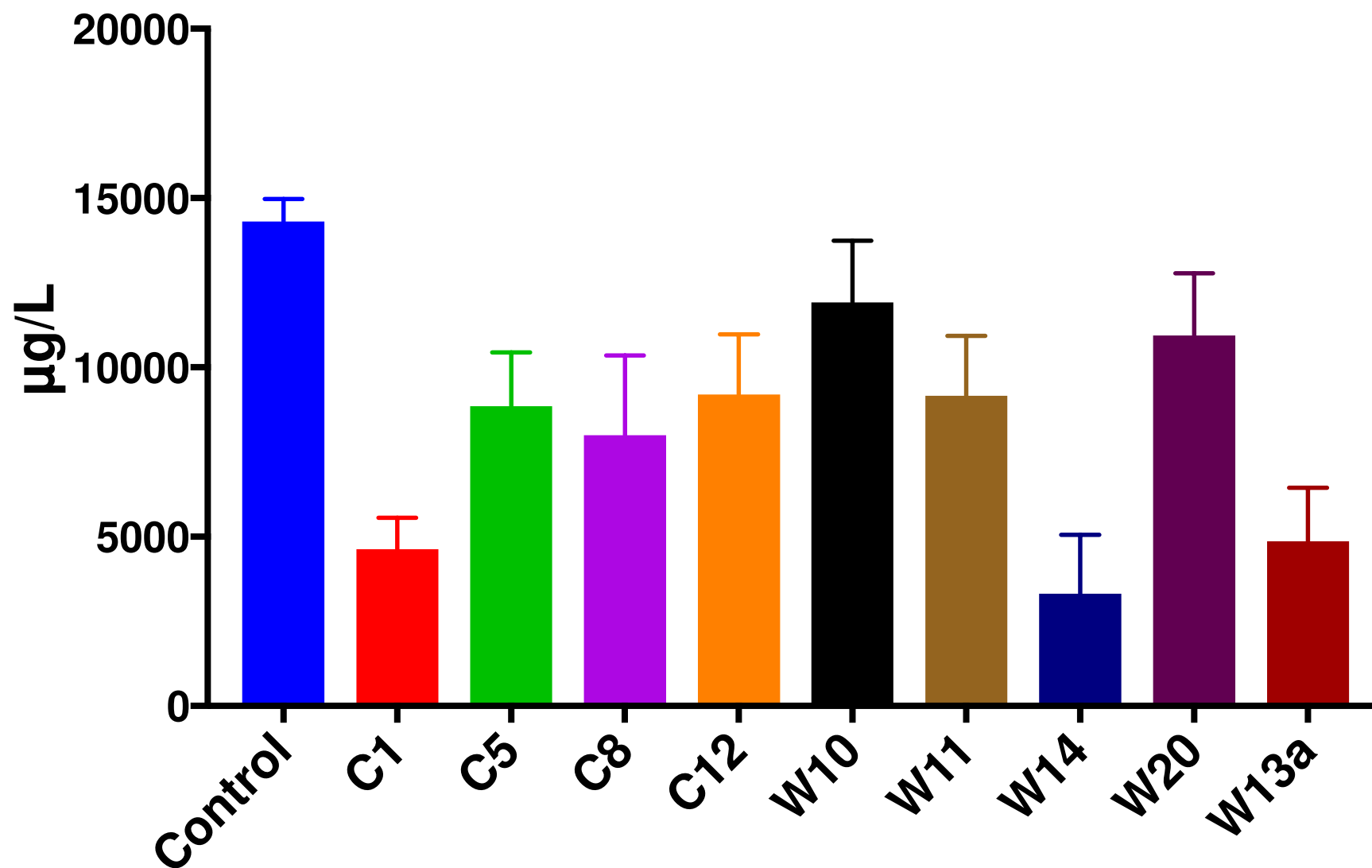

Supplement: S4 Fig — Error bars represent the standard deviation of three replicates. (PDF) [file pone.0208406.s004.pdf]

**S5 Fig.**

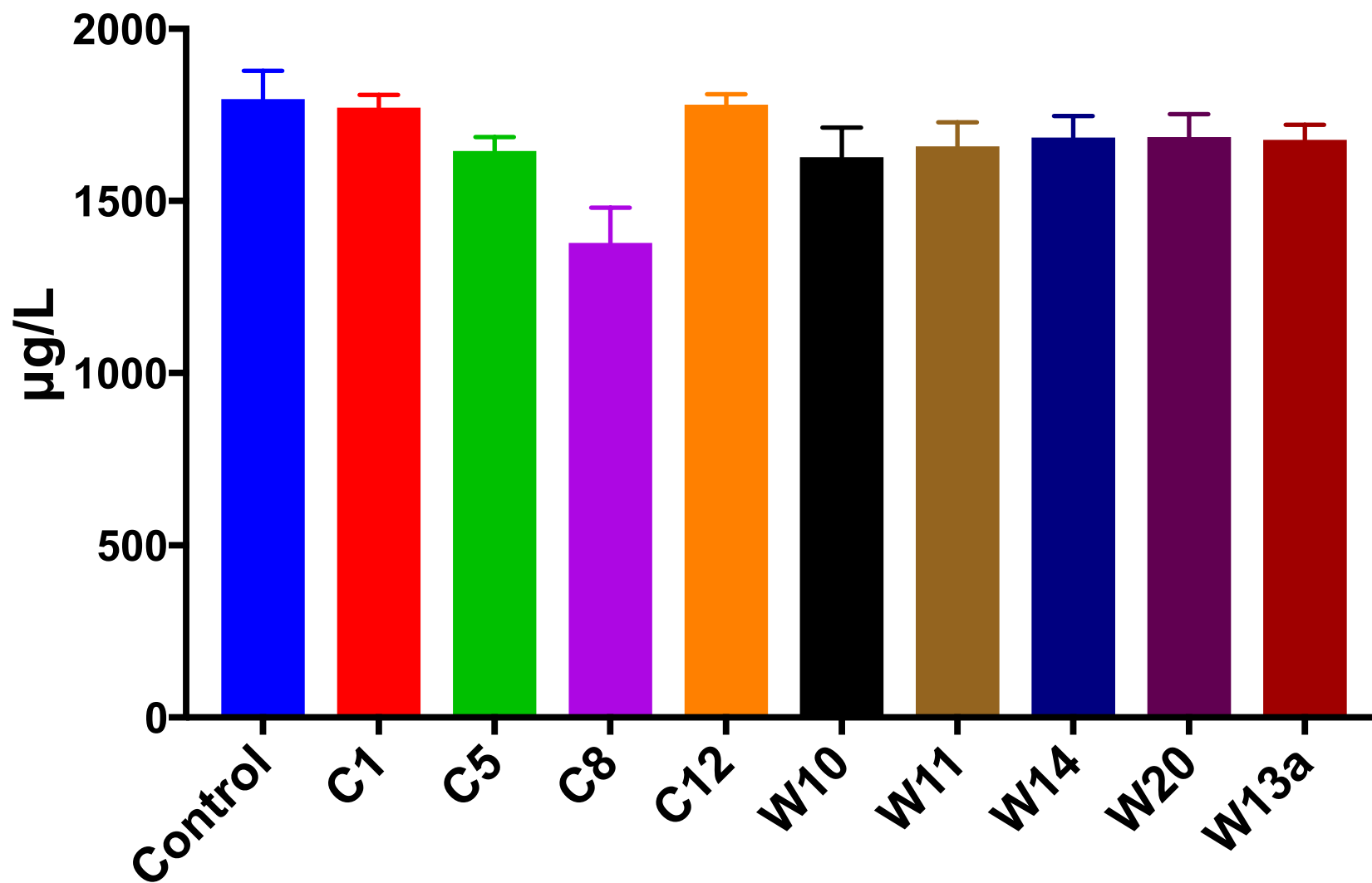

Supplement: S5 Fig — Error bars represent the standard deviation of three replicates. (PDF) [file pone.0208406.s005.pdf]
